# Resolving Contributions of Oxygen-Consuming and ROS-Generating Enzymes at the Synapse

**DOI:** 10.1155/2016/1089364

**Published:** 2016-11-27

**Authors:** Engy A. Abdel-Rahman, Ali M. Mahmoud, Abdullah Aaliya, Yasmine Radwan, Basma Yasseen, Abdelrahman Al-Okda, Ahmed Atwa, Eslam Elhanafy, Moaaz Habashy, Sameh S. Ali

**Affiliations:** Center for Aging and Associated Diseases, Helmy Institute of Medical Sciences, Zewail City of Science and Technology, Giza, Egypt

## Abstract

Disruption of cellular redox homeostasis is implicated in a wide variety of pathologic conditions and aging. A fundamental factor that dictates such balance is the ratio between mitochondria-mediated complete oxygen reduction into water and incomplete reduction into superoxide radical by mitochondria and NADPH oxidase (NOX) enzymatic activity. Here we determined mitochondrial as well as NOX-dependent rates of oxygen consumption in parallel with H_2_O_2_ generation in freshly isolated synaptosomes using high resolution respirometry combined with fluorescence or electrochemical sensory. Our results indicate that although synaptic mitochondria exhibit substantially higher respiratory activities (8–82-fold greater than NOX oxygen consumption depending on mitochondrial respiratory state), NADPH-dependent oxygen consumption is associated with greater H_2_O_2_ production (6-7-fold higher NOX-H_2_O_2_). We also show that, in terms of the consumed oxygen, while synaptic mitochondria “leaked” 0.71% ± 0.12 H_2_O_2_ during NAD^+^-linked resting, 0.21% ± 0.04 during NAD^+^-linked active respiration, and 0.07% ± 0.02 during FAD^+^-linked active respiration, NOX converted 38% ± 13 of O_2_ into H_2_O_2_. Our results indicate that NOX rather than mitochondria is the major source of synaptic H_2_O_2_. The present approach may assist in the identification of redox-modulating synaptic factors that underlie a variety of physiological and pathological processes in neurons.

## 1. Introduction

Substantial evidence indicates that the synapse is a center stage for brain physiology and pathology [1]. Synaptic activity is now known to produce ROS that are essential regulators of multitudes of normal physiological processes in neurons including cognition and memory. The high levels of ROS generation in synapses, alongside their high-energy demands, make them more vulnerable to stressful insults encountered in aging, neurodegenerative, neuropsychological, and neurodevelopmental disorders [2]. The relative importance of specific enzymatic sources of ROS in synapses is not fully understood. Mitochondria are one source of cellular ROS. A portion of oxygen consumed by mitochondria escapes the aerobic ATP production pathway and forms oxygen radicals primarily in the form of superoxide anions (O_2_^•−^) that are instantaneously dismutated to hydrogen peroxide (H_2_O_2_) by mitochondrial superoxide dismutase (SOD) [3–5]. Since the brain is highly metabolically active organ that exhibits robust oxygen consumption [6], mitochondria respiratory activity was often considered the prime source of brain ROS. However, recent data indicate that NADPH oxidases (NOX), the enzyme family known to generate ROS as their only and primary function, are widely expressed in the CNS where they considerably contribute to ROS generation [7, 8]. While NOX2 is found principally in phagocytes, recent reports showed that NOX2 and homologs (NOX1, NOX3–5, Duox1, and 2) are expressed in a miscellaneous array of tissues and cell types. NOX2 and NOX4 have been characterized in the neurons of adult mouse nervous system, potentially contributing to wide range of physiologic functions and to several neurological disorders [9]. Although it is likely that the initial product of all NOX enzymes is O_2_^•−^, which spontaneously dismutates to H_2_O_2_ *via* superoxide dismutase (SOD), it is now clear that H_2_O_2_ is predominantly produced by several NOX isoforms, particularly NOX4, Duox1, and Doux2. This apparent H_2_O_2_ generation may be attributed to the rapid dismutation of O_2_^•−^. However, recent reports showed that for, NOX4, H_2_O_2_ generation is mediated by the third extracellular loop of the enzyme (reviewed in [10]).

Synaptic localization of mitochondria [11, 12] and some NOX isoforms have been documented [8, 13]. Synaptosomes (isolated nerve terminals), which have been extensively used for studying brain synaptic physiology, were found to contain mitochondria with distinct biophysical properties from those of neuronal body mitochondria [11, 12]. NOX2 and NOX4 are also expressed in synaptosomal plasma membrane [8, 13] and we previously reported that synaptosomes exhibit NADPH-dependent oxygen consumption [13]. Given the critical role of NOX and mitochondria in cellular ROS production and the presence of some NOX isoforms and distinctive mitochondria in synaptosomes, it is of paramount importance to characterize the interplay between their respiratory functions and ROS generation in synapses. Our previous study using spin-trapping electron paramagnetic resonance spectroscopy showed that NOX rather than mitochondria was the main contributor of synaptic superoxide generation [8]. However, the relationship between mitochondria and/or NOX-dependent O_2_ consumption and resulting ROS generation is still ambiguous, despite the long-held idea that augmented energy expenditure will result in higher ROS generation [14]. Much vagueness exists over the relationship between the rate of oxygen consumption and the production of reactive oxygen species (ROS) such as hydrogen peroxide by mitochondria and NOX in synapses. Here, we use combined high resolution respirometry and fluorometry to simultaneously monitor oxygen consumption and H_2_O_2_ production by synaptosomal mitochondria and NOX. We will describe for the first time how the well-established Amplex Red assay or an electrochemical sensor can be used to quantify H_2_O_2_ production by NOX combined with the simultaneous measurement of NOX-dependent oxygen consumption by high resolution respirometry.

## 2. Materials and Methods

### 2.1. Animals

C57BL6/C males (6 weeks old) were purchased from Misr University for Science and Technology (Cairo, Egypt) and were housed for at least one month in Zewail City animal facility until sacrificed. All animals were maintained in pathogen-free, individually ventilated cages in 12 h light/12 h dark cycles at 24°C and 50% relative humidity, with free access to water and standard laboratory rodent chow. Animals were decapitated following quick cervical dislocation which is an approved and considered humane method of small animal euthanasia by the American Veterinary Medical Association (AVMA) (https://www.avma.org/KB/Policies/Documents/euthanasia.pdf). All experiments were conducted in adherence to the NIH Institutional Animal Care and Use Committee guidelines https://grants.nih.gov/grants/olaw/GuideBook.pdf.

### 2.2. Isolation of Synaptosomes

Isolation of synaptosomes was performed as previously described [13]. Briefly, brains were quickly removed; forebrains were dissected and homogenized using a Dounce homogenizer in ice-cold isolation buffer (0.32 M sucrose, 1 mM EDTA, 10 mM Tris-HCl buffer, pH 7.4, 10 mM glucose). The homogenate was then centrifuged at 3,100 ×g for 3 min at 4°C. The supernatant was removed and the pellet was resuspended in half the volume of isolation buffer, then homogenized again, and recentrifuged. The supernatant was collected and mixed with percoll to a final concentration of 15% by volume. The mixture was then layered onto a step gradient of 23% and 40% percoll. Centrifugation was then performed at 16,000 rpm for 5 min at 4°C. The band at the interface of the two layers was collected and rinsed in isolation buffer, followed by centrifugation and resuspension in synaptosomal buffer (120 mM NaCl, 4.7 mM Kcl, 2.2 mM CaCl_2_, 1.2 mM MgCl_2_, 25 mM HEPES, 1.2 mM MgSO_4_, 1.2 mm KH_2_PO_4_, and 10 mM glucose).

### 2.3. Determination of NOX Activity in Synaptosomes by Oroboros® High Resolution O2k Oxygraph

NOX activity in synaptosomal preparations was determined by measuring, in the same sample, NADPH-induced oxygen consumption and the associated rate of hydrogen peroxide formation simultaneously using Amplex Red fluorescence/or H_2_O_2_ electrochemical HPO-ISO-2 mm sensor (WPI, Sarasota, USA) which is compatible with the O2k-NO Amp-Module (Oroboros®).

### 2.4. Measurements of NADPH Oxidase Respiratory Rates

NADPH oxidase respiratory assessments (and hydrogen peroxide determinations) were carried out at 37°C using the high resolution respirometry system Oxygraph-2K (Oroboros Instruments, Innsbruck, Austria) in 2 mL chambers. Before starting the experiment, calibration at air saturation was performed by allowing the respiration medium, MIR05, to equilibrate with air in the oxygraph chambers and be stirred at 540 to 560 rpm for 30 to 40 min, until a stable signal was detected. Synaptosomal protein (0.2 mg) was added to the respiration medium in the chamber. Activation of NOX was evoked by the addition of 200 *μ*M NADPH (3 doses). The rates of oxygen consumption were calculated as the negative time derivative of oxygen concentration. The rate of NOX-dependent hydrogen peroxide formation was detected in parallel to oxygen consumption in the same sample by using two different approaches. In the fluorometric method, horseradish peroxidase (1 U per mL) and Amplex UltraRed fluorescent dye (10 *μ*M) were utilized. The excitation wavelength was 525 nm and fluorescence detection was at 587 nm. For determination of NOX-dependent hydrogen peroxide production by amperometric detection in parallel with oxygen consumption, the fluorescent dye was substituted by inserting an HPO-ISO-2 WPI electrode in the O2k chamber. Signals were calibrated using known amounts of hydrogen peroxide that were exogenously added by the end of each run. At the peak of oxygen-consuming, HPO-producing NADPH activity, specific NOX inhibitor VAS-2870 (10 *μ*M) or ebselen (10 *μ*M) was added to confirm that the activity recorded was mediated by NOX. Data acquisition and analysis were performed with the DatLab® software, version 4.3 (Oroboros Instruments). This enables continuous monitoring and recording of the oxygen concentration in the chambers as well as of the derived oxygen flux over time, normalized for the amount of homogenized tissue acquired at rates of 0.5–1 Hz.

### 2.5. Measurements of Synaptic Mitochondrial Oxygen Consumption and Hydrogen Peroxide Production

After blocking NOX activity, synaptosomes were permeabilized by the addition of saponin (25 *μ*g/mL). State 4 respiration was triggered by adding the following substrates: 10 mM pyruvate + 10 mM malate + 10 mM glutamate. State 3 respiration was then induced by adding ADP (1 mM) for measuring OXPHOS I followed by 10 mM succinate to assess OXPHOS I+II. Parallel assessment of oxygen consumption and hydrogen peroxide formation by synaptosomal mitochondria was performed as described above. In a control experiment, we confirmed that outer mitochondrial membrane integrity was not affected by the saponification process as exogenously added cytochrome c (10 *μ*M) did not impact mitochondrial OCR (data not shown).

## 3. Results

### 3.1. NADPH Oxidases Are Minor Oxygen Consumers but Major Hydrogen Peroxide Producers in Synaptosomes

**Figure 1:**
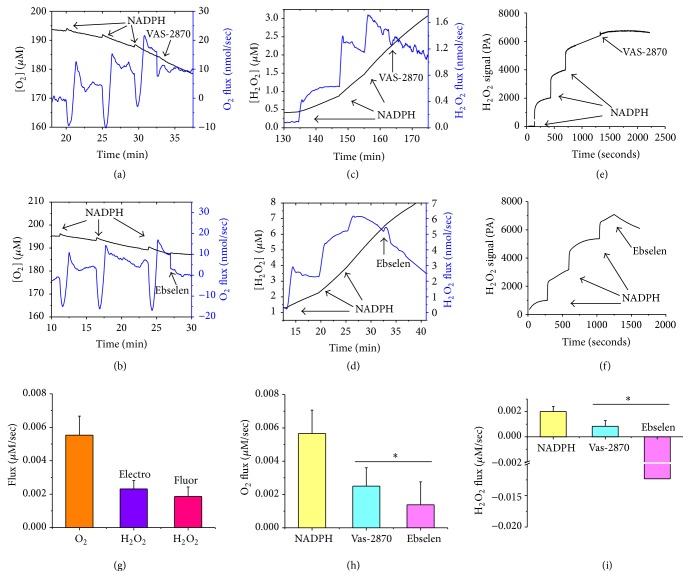
NADPH oxidases are actively consuming O_2_ and producing H_2_O_2_ in freshly isolated synaptosomes. Successive injections of 200 *μ*M deoxygenated NADPH batches on synaptosomes triggered reproducible oxygen consumption (a, b) and H_2_O_2_ production as detected simultaneously by HRP/Amplex Red fluorescence (c, d) or using electrochemical sensor (e, f). The effects of inclusion of the NOX inhibitors Vas-2870 (10 *μ*M) (a, c, e) or ebselen (10 *μ*M) (b, d, f) are shown. (g) Quantifications of the overall O_2_ and H_2_O_2_ fluxes induced by the three added doses of NADPH. The two methods of H_2_O_2_ detection yielded similar results. Both of the added NOX inhibitors, VAS-2870 (10 *μ*M) and Ebselen (10 *μ*M), caused significant inhibition of NADPH-triggered oxygen flux (h) and H_2_O_2_ flux (i). Values are given as mean ± SEM, paired Student *t*-test was used for paired groups to determine statistical significance in comparison with NADPH-induced activity, ^*∗*^ *p* < 0.05, and *n* = 5–8.

We have previously reported the detection of oxygen consumption by synaptosomal NOX using Oxygraphy [8, 13]. Here, we utilized high resolution respirometry (Oroboros O2k-Station) to follow NADPH-induced oxygen consumption in isolated C57BL6/C male synaptosomes. Additionally, we simultaneously used the O2k-Fluorometer or the HPO sensor, for the first time, to monitor the rate of hydrogen peroxide production in the same sample. In the fluorometric assay the highly sensitive AR/HRP system has been used for assessment of H_2_O_2_ production by recording changes in resorufin fluorescence [15]. Since HRP has been shown to catalyze the oxidation of NADPH with subsequent generation of H_2_O_2_ [16], we compared NOX-dependent oxygen consumption and hydrogen peroxide production obtained by using the AR/HRP system and the HPO sensor. In Figures 1(a)–1(f), we show representative traces of NADPH-induced, VAS-2870-inhibitable (a, c, e), or ebselen-inhibitable (b, d, f) synaptosomal oxygen consumption (a, b) and parallel hydrogen peroxide production obtained using AR/HRP fluorometric assay (c and d), or Oroboros-compatible amperometric HPO sensor (e, f). Using the AR/HRP assay, we could continuously monitor H_2_O_2_ generated during NOX activation. We calibrated our H_2_O_2_ fluorescence signal using high resolution measurements of oxygen production due to selective decomposition of H_2_O_2_ by exogenous catalase (not shown). This allowed us to quantify the O_2_ proportions that are converted to H_2_O_2_ in real time. Previous studies showed that NADPH can generate H_2_O_2_ nonenzymatically through its interaction with HRP [17]. In this regard, we found that, in the absence of added synaptosomes, the addition of NADPH alone in our AR/HRP assay resulted in enhanced fluorescence. However, under our experimental conditions, this increase in background fluorescence was far less than the resorufin fluorescence detected in the presence of synaptosomes. Our results are in tune with another study utilizing the AR/HRP system for microsomal enzymes activity, which showed that increased resorufin fluorescence resulting from the interaction between HRP and NADPH was less than 2–5% of the fluorescence monitored in the presence microsomal enzymes [17].

After subtraction of background fluorescence, there were no significant differences in oxygen consumption by synaptosomal NOX between fluorometric (*n* = 5) and electrochemical (*n* = 5) experiments. As shown in Figure 1(g), the hydrogen peroxide production by synaptosomal NOX did not significantly differ between the two experimental approaches. Using O2k combined with the AR/HRP fluorescence module, the addition of 200 *μ*M NADPH (3 doses) to synaptosomal proteins (0.2 mg) in our study resulted in absolute rates of oxygen consumption and H_2_O_2_ production of 0.0055 ± 0.0011 *μ*M/s and 0.0018 ± 0.0005 *μ*M/s H_2_O_2_. Similar rates of H_2_O_2_ production were obtained when the AR/HRP system was replaced by HPO electrode 0.0023 ± 0.0005 *μ*M/s H_2_O_2_, confirming that there was minor or no interference from components of the reaction mix.

Both VAS-2870 and ebselen significantly inhibited NADPH-induced oxygen consumption by about 50–75% (*N* = 5–8, *p* < 0.05). However, while VAS-2870 reduced H_2_O_2_ production proportionately by ~60% (*N* = 8, *p* < 0.05), we observed a strong elimination of hydrogen peroxide by ebselen, which is in tune with reports showing that the later possesses glutathione peroxidase activity (reviewed in [18]).

### 3.2. Relative to NOX, Mitochondria Are Major Oxygen Consumers but Minor Hydrogen Peroxide Producers in Synaptosomes

**Figure 2:**
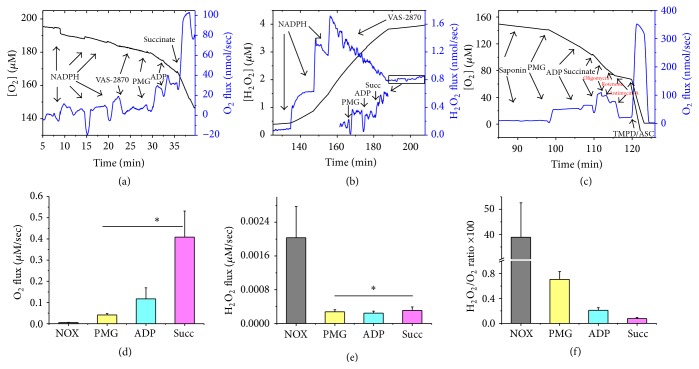
Comparing NADPH oxidase and mitochondrial activities in isolated synaptosomes using high resolution respirometry and fluorescence spectroscopy. (a, b) Representative traces of NADPH oxidase and synaptosomal mitochondria activity assessed by simultaneous measurement of rates of oxygen consumption (a) and H_2_O_2_ production (b) using high resolution respirometry combined with HRP/Amplex Red, in the same sample under identical conditions except that NOX substrate is substituted by mitochondria ones. Activities were monitored as described in Figure 1's legend. Mitochondrial resting (state 4) respiration was triggered by the addition of 10 mM pyruvate + 10 mM malate + 10 mM glutamate. Active phosphorylating (state 3) respiration was induced by adding 1 mM ADP (OXPHOS I) followed by 10 mM succinate for (OXPHOS I+II). (c) Representative traces depicting ETC substrate-specific O_2_ utilization by mitochondria in saponin-permeabilized synaptosomes during substrate-uncoupler inhibitor-titration. (d, e) Quantifications of the sum of the absolute values of O_2_ (d) and H_2_O_2_ (e) fluxes following NOX and mitochondria activations. (f) Percept ratios of the H_2_O_2_-produced to the O_2_-consumed. Values are given as mean ± SEM, paired Student *t*-test was used to determine statistical significance in comparison with NADPH-induced collective activity, and *n* = 7. ^*∗*^ *p* < 0.05.

To investigate the relative importance of NOX and mitochondria in energy expenditure and H_2_O_2_ production at the synapse, we employed isolated nerve terminals (synaptosomes) that contain both pre- and postsynaptic vesicles. Synaptosomal preparations are populated with NOX isoforms and mitochondria of characteristic biophysical properties. Experiments typically involved parallel measurements of oxygen consumption and hydrogen peroxide production using high resolution respirometry combined with the HRP/Amplex Red fluorescence assay. This approach allows one to follow both NOX and mitochondrial activities in synaptosomes and estimate resultant hydrogen peroxide production in the same sample. When NADPH was added to synaptosomes, oxygen consumption and hydrogen peroxide production due to NOX activity were triggered as described above. This activity was abolishable by the specific NOX inhibitor VAS-2870 (10 *μ*M) or ebselen (10 *μ*M). Subsequent addition of pyruvate, malate, and glutamate to synaptosomes elicited oxygen consumption due to NAD^+^-linked resting mitochondrial metabolic activity (Figure 2(a)) while also “leaking” hydrogen peroxide in parallel (Figure 2(b)). In a separate experiment, we confirmed the involvement of synaptosomal mitochondria in the substrate-triggered oxygen consumption (Figure 2(c)). As can be seen in Figure 2(c), both NAD^+^- and FAD^+^-linked respiratory activities were completely inhibitable by oligomycin (complex V), rotenone (complex I), and Antimycin A (complex III) which demonstrate the presence of viable mitochondria within the prepared synaptosomes. Quantifications of the results in Figures 2(a) and 2(b) over *n* = 7 independent synaptosomal preparations are given in Figures 2(d) and 2(e). It can be readily seen that NOX-mediated hydrogen peroxide production was 38.85% ± 13 of NOX-dependent oxygen consumption detected in the same samples (Figure 2(f)). In contrast, synaptosomal mitochondrial oxygen consumptions during state 4, state 3 (OXPHOS I), and state 3 (OXPHOS I+II) were only associated with 0.71% ± 0.12, 0.20% ± 0.04, and 0.075% ± 0.020, H_2_O_2_ production, respectively (Figure 2(f)). Additionally, Figure 2(d) shows that the rates of oxygen consumption during state 4 (*Leak I*), state 3 (OXPHOS I), and state 3 (OXPHOS I+II) in synaptosomal mitochondria are significantly higher than NOX-dependent oxygen consumption rate in synaptosomes (0.041 ± 0.007 *μ*M/s, 0.117 ± 0.050 *μ*M/s, 0.408 ± 0.120 *μ*M/s, 0.005 ± 0.002 *μ*M/s, resp., for mitochondria, and NOX, *n* = 7). However, Figure 2(e) shows that NOX-dependent H_2_O_2_ production rate in synaptosomes is higher than H_2_O_2_ generated during state 4 (*Leak I*), state 3 (OXPHOS I), and state 3 (OXPHOS I+II) by synaptosomal mitochondria (0.0020 ± 0.0007 *μ*M/s; 0.00030 ± 0.00005 *μ*M/s; 0.00025 ± 0.00005 *μ*M/s; 0.00030 ± 0.00008 *μ*M/s, resp., for NOX and mitochondria *n* = 7, *p* < 0.05).

## 4. Discussion

Probing the relation between oxygen consumption and ROS production makes it possible to understand how specific ROS sources might shape cellular physiology and pathology [19, 20]. The use of the highly stable Clark-type oxygen electrode in the Oroboros O2k Station with its superb picomole sensitivity in addition to the fluorescence module designed to follow H_2_O_2_ in the same sample has enabled the interrogation of each mitochondrial respiratory state while quantifying ROS leakage simultaneously [15]. We now demonstrate that this approach can also be used successfully for parallel monitoring of NOX-dependent oxygen consumption as well as H_2_O_2_ production in synaptosomes. Our findings reveal for the first time a negative correlation between synaptic energy utilization and ROS production and provide evidence that synaptosomal NOX consumes significantly less oxygen while producing remarkably more synaptic H_2_O_2_. However, synaptosomal mitochondria were more effectively utilizing oxygen while producing smaller ROS amounts.

We have previously reported a synaptic localization of NOX2 and NOX4 isoforms as well as NOX-dependent oxygen consumption in synaptosomes [13]. In the present study, we describe the use of a highly sensitive assay using AR/HRP and an electrochemical approach to quantify H_2_O_2_ production by NOX in synaptosomes combined with the simultaneous measurement of NOX-dependent oxygen consumption by high resolution respirometry. In mammalian cells, H_2_O_2_ was found to play a fundamental role in the redox regulation of several physiological and pathological processes. Although it is likely that H_2_O_2_ arises as a major by-product of mitochondrial respiratory activity with complex I being the main source of mitochondrial ROS [21], it has been increasingly recognized that NADPH oxidase is a key source of cellular ROS. Among the ROS generated, H_2_O_2_ is produced by NOX activity [22]. Despite the fact that the rate of ROS generation is not governed by the oxygen availability in its physiological range, ROS are produced as a consequence of oxygen consumption. It is therefore of fundamental importance to resolve how much of the total oxygen consumed by NOX and mitochondria are directed toward ROS formation. In this context, we used the high resolution O2k sensor combined with AR/HRP system to follow the relative contributions of mitochondria and NOX to the process of hydrogen peroxide generation in synaptosomes.

Our study revealed that NOX considerably contributes to the levels of hydrogen peroxide in synaptosomes. However, the higher level of NOX-dependent H_2_O_2_ was associated with a lower oxygen consumption rate. We also found that 38.85% of oxygen consumed by synaptic NOX is converted to H_2_O_2_. The initial product of all NOX enzymes is O_2_^•−^, which spontaneously dismutates to H_2_O_2_, (reviewed in: [10]). H_2_O_2_ is predominantly detected for several NOX isoforms, particularly NOX4, Duox1, and Doux2. This apparent direct H_2_O_2_ generation may be attributed to the rapid dismutation of O_2_^•−^. The ~0.4 stoichiometry of H_2_O_2_ formation relative to NOX-dependent oxygen consumption obtained in our study is in accordance with the previously suggested superoxide dismutase-like mechanism involving two oxygen binding/reduction steps for every H_2_O_2_ generated in NOX4 active site [23]. This is inconsistent with a proposed mechanism involving single oxygen molecule binding, followed with reduction by heme center in two sequential electron transfer steps, to produce superoxide intermediate [24]. Therefore, while either mechanism could participate in the generation of small amounts of superoxide [24], our results are in agreement with a mechanism involving two oxygen binding/reduction steps. However, since it is not possible to dismiss that the detected H_2_O_2_ is resulting from the dismutation of O_2_^•−^ by synaptosomal SOD, both NOX2 and NOX4 might be contributing to the generation of NADPH-induced H_2_O_2_ signals. We attempted to disentangle NOX isoforms contributions using the established NOX inhibitors VAS-2870 (preassembled NOX2 and NOX4 inhibitor) and ebselen (proposed as a potent NOX2 inhibitor), recently reviewed in [25]. Interestingly, 10 *μ*M VAS-2870 inhibited ~50% of NADPH-induced activity whether it is recorded as oxygen consumption or as H_2_O_2_ production (Figures 1(h) and 1(i)). Meanwhile, 10 *μ*M ebselen quenched ~75% of NADPH-induced oxygen consumption while completely reversing H_2_O_2_ signal (Figures 1(h) and 1(i)). This is consistent with previous reports that selenium-containing ebselen is able to consume hydrogen peroxide in a catalytic cycle that utilizes thiol-containing compounds, such as glutathione, as a substrate (reviewed in [18]). Although not sufficient to quantify individual contributions, these results indicate that both NOX2 and NOX4 are important contributors to the observed NADPH-induced activities in synaptosomes.

Finally, we evaluated in the same synaptosomal sample the proportion of the mitochondrially utilized oxygen that converts into hydrogen peroxide during complex I-mediated resting respiration and complex I and complex I+II-mediated active respiration. Mitochondria residing at synapses play crucial role in synaptic function and failure. Synaptic mitochondria have biophysical properties that are distinct from that of their siblings in the soma [11, 12]. Our results revealed that only 0.71% of oxygen consumed by synaptic mitochondria during complex I resting respiration was converted to H_2_O_2_, which is not markedly different from that previously reported for isolated rat brain mitochondria. That is, previous report showed that 0.79% percent of the total oxygen consumption by isolated rat brain mitochondria produced hydrogen peroxide during complex I resting respiration [26]. The underlying mechanism of the observed low rates of hydrogen peroxide production by mitochondria compared to NOX may involve a respiratory protection conferred by slips (intrinsic decoupling) in mitochondrial redox proton pumps. In fact, consensus from several studies demonstrating that intrinsic decoupling between the flow of electrons and proton translocation prevents excessive electronegativity of redox carriers in complexes I and III, which lowers free [O_2_] and retards the generation of O_2_^•−^. In addition, respiratory protection conveyed by “mild” uncoupling that is caused by H^+^ leakage across the mitochondrial membrane could contribute to the observed lower rates of mitochondrial hydrogen peroxide production in synaptosomes. In line with this reasoning, it has been shown that a minor reduction in ΔΨ, due to mild uncoupling, would, in fact, prevent O_2_^•−^ formation. Therefore intrinsic decoupling and mild uncoupling were suggested to have a natural antioxidant effect, contributing to lower rates of mitochondrial hydrogen peroxide production [reviewed in [27]]. No previous study has investigated the relationship between the rate of oxygen consumption and the production of reactive oxygen species (ROS) such as hydrogen peroxide by synaptosomal mitochondria and NOX. Therefore, we believe that this is the first report that addressed in detail the interplay between synaptic ROS generation by NADPH oxidases and mitochondria and their respiratory functions.

## 5. Conclusion

We employed high resolution respirometry equipped with a fluorescence detection module to simultaneously monitor oxygen consumption and H_2_O_2_ production by NADPH oxidase and mitochondria in synaptosomes. Using this assay, we showed that NOX consumes less oxygen and produces more ROS, contributing considerably to synaptic H_2_O_2_ generation. However, mitochondria at synapses were utilizing oxygen more efficiently while producing smaller ROS amounts. Our results may eventually assist in understanding the synaptic mechanisms by which specific ROS sources are implicated in neuronal physiological as well as pathological processes.
