# Corrigendum to “Resolving Contributions of Oxygen-Consuming and ROS-Generating Enzymes at the Synapse”

**DOI:** 10.1155/2017/1710632

**Published:** 2017-12-28

**Authors:** Engy A. Abdel-Rahman, Ali M. Mahmoud, Abdalla G. Alia, Yasmine Radwan, Basma Yasseen, Abdelrahman AlOkda, Ahmed Atwa, Eslam Elhanafy, Moaaz Habashy, Sameh S. Ali

**Affiliations:** Center for Aging and Associated Diseases, Helmy Institute of Medical Sciences, Zewail City of Science and Technology, Giza, Egypt

In the article titled “Resolving Contributions of Oxygen-Consuming and ROS-Generating Enzymes at the Synapse” [1], the names of the third and sixth authors were given incorrectly as Abdullah Aaliya and Abdelrahman Al-Okda. The authors' names should have been written as Abdalla G. Alia and Abdelrahman AlOkda. The revised authors' list is shown above.
